# Supplementary material for: Exploring the Effectiveness of Problem-Based Learning in an International Undergraduate Program in Veterinary Sciences: Students’ Satisfaction, Experience and Learning
Source: Vet Sci. 2024 Feb 27;11(3):104. doi: 10.3390/vetsci11030104 (PMC10974726; doi:10.3390/vetsci11030104)
Supplement: Supplementary file 1 [file vetsci-11-00104-s001.zip › Supplementary Materials S1-S6.pdf]

**Table S1. List of expected learning outcomes for each learning objective of the PBL module**

| Learning Objective | Learning Outcome |
|---|---|
| definition of zoonosis | Define zoonosis;<br><br>Understand the ways of infectious disease transmission between humans and animals and vice versa. |
| identification of symptoms and routes of transmission of selected zoonotic diseases | Recognize the signs and symptoms of Mycobacteriosis and Toxoplasmosis;<br><br>Describe the pathways of transmission of Mycobacteriosis and Toxoplasmosis. |
| principles of biosecurity in zoological gardens | Experience the principles of biosecurity applied in zoological gardens;<br><br>Wear properly personal protective equipment;<br><br>Properly sample, package, store and ship biological materials. |
| use of environmental enrichments | Identify the different types of environmental enrichments;<br><br>Discuss the characteristics enrichments must have to be efficient and feasible;<br><br>Apply the SPIDER acronym when implementing an enrichment program. |
| social organization and management of captive animals | Identify the main features that describe social systems in animals;<br><br>Understand the complexity and the plasticity of animal social lives and advantages and disadvantages of group living;<br><br>Evaluate and discuss the implications of the specie’s social system for the care of animals under human management. |
| Physiological variables of respiratory gas exchange | Understand the intimate relationships between simply observable physiological variables, such as ventilation rate (VR) and heat rate (HR) in mammals, and oxygen delivery to tissues;<br><br>Understand why VR and HR can be indicative of animal health and diseases;<br><br>Recognize pathological and physiological respiratory patterns in animals. |
| the morphology of the blood cells and the tuberculoid granuloma | Describe and understand the physiological significance of selected hematological parameters (gross morphology of blood cells, hematocrit and leukogram); |

| | |
|---|---|
| | Understand the role of white blood cells in forming granuloma as a reaction to infections, inflammation, irritants or foreign objects. |
| communication skills | Identify the key elements in the communication process;<br><br>Understand the importance of a proper communication in critical situations. |

**Table S2. Self and peer evaluation.**

| Question | 4=excellent | 3=good | 2=fair | 1=poor |
|---|---|---|---|---|
| She/he participates in group work | | | | |
| She/he contributes to the success of the project | | | | |
| She/he listens to others | | | | |
| She/he asks and answers questions | | | | |
| She/he stays on the task | | | | |
| She/he finds and provides quality information | | | | |
| She/he cooperates with others | | | | |
| She/he offers positive suggestions | | | | |
| She/he shows leadership | | | | |
| She/he compliments and encourages others | | | | |

**Table S3. Tutor Assessment tool**

The tutor evaluates the student's overall behavior during all tutoring activities assigned to her/him. The tutor grades the student's behavior using the following letters.

The student:

A = always shows this behavior at a high level of performance.

B = shows this behavior at a good level of performance.

C = shows this behavior at a qualitatively sufficient level of performance.

D = shows this behavior insufficiently.

| Question | A | B | C | D |
|---|---|---|---|---|
| *Problem analysis skills* | | | | |
| Contributes to the problem analysis | | | | |
| Expresses own ideas about the problem (brainstorming) | | | | |
| Formulates hypotheses | | | | |
| Discusses hypotheses made by others | | | | |
| Formulates learning objectives | | | | |
| Justifies own hypotheses | | | | |
| *Problem-solving ability* | | | | |
| Communicates her/his bibliographic sources to the group | | | | |

| | | | | |
|---|---|---|---|---|
| Tests hypotheses in the light of new knowledge | | | | |
| Communicates what she/he has studied to others in a clear and orderly manner | | | | |
| Uses the knowledge acquired in problem-solving | | | | |
| Expresses her/his opinion during the evaluation of group work | | | | |
| *Collaboration skills* | | | | |
| Plays the role of group moderator/scribe/secretary appropriately | | | | |
| Participates in the discussion | | | | |
| Listens to the opinions of others | | | | |
| Provides constructive criticism | | | | |
| Accepts the criticisms from others | | | | |
| Expresses herself/himself clearly | | | | |
| Cooperates and helps others | | | | |
| Contributes to the good functioning of the group | | | | |
| *Comments and suggestions* | | | | |

**Table S4. Module and Problem evaluation**

Using the following scale:

5. I completely agree with the statement

4. I agree, with some reservations

2. I somewhat disagree

1. I completely disagree.

Answer the questions about the MODULE by indicating your degree of agreement or disagreement.

| Question | 5 | 4 | 2 | 1 |
|---|---|---|---|---|
| Considering the module as a whole, I worked with pleasure | | | | |
| The content of the module was suitable for my prior knowledge | | | | |
| The content of the module was easy to understand | | | | |
| I learned a lot from this module | | | | |
| I consider the topic of this module very important | | | | |
| The module was very well planned | | | | |
| The module was carried out harmoniously | | | | |

Answer the questions about the PROBLEMS by indicating your degree of agreement or disagreement.

| Question | 5 | 4 | 2 | 1 |
|---|---|---|---|---|
| The problems were clearly expressed | | | | |
| The problems were appropriate for the systematic use of problem-based learning | | | | |
| The problems sufficiently stimulated group discussion | | | | |

| | | | | |
|---|---|---|---|---|
| The problems were satisfactorily fitting to the objectives of the module | | | | |
| The problems sufficiently stimulated independent learning | | | | |
| The problems helped me to integrate the various disciplines | | | | |
| During this module I learned many other things that were not related to the problems themselves | | | | |
| The problems appeared to be sufficiently varied to me | | | | |

Answer the questions about the GROUP PROCESS by indicating your degree of agreement or disagreement.

| Question | 5 | 4 | 2 | 1 |
|---|---|---|---|---|
| My group applied the steps of problem-based learning in a systematic way | | | | |
| My group agreed explicitly to comply with the study topics | | | | |
| Each student usually respected his/her study commitment | | | | |
| Everyone participated actively in the discussion | | | | |
| Tutorial sessions were very productive | | | | |
| The meetings stimulated my independent learning activities | | | | |
| The atmosphere in my group was pleasant | | | | |

**Table S5. Tutor evaluation**

Using the following scale:

5. I completely agree with the statement

4. I agree, with some reservations

2. I somewhat disagree

1. I completely disagree.

Answer the questions about the TUTOR by indicating your degree of agreement or disagreement.

| Question | 5 | 4 | 2 | 1 |
|---|---|---|---|---|
| The tutor was familiar with the principles of problem-based learning | | | | |
| I had the impression that the tutor liked her/his role | | | | |
| The tutor encouraged us to work hard | | | | |
| The tutor's questions stimulated discussion | | | | |
| The tutor regularly evaluated with us how the group was performing | | | | |
| The tutor seemed interested in the personal and social integration of each student in the group | | | | |
| The tutor gave me frequent feedback | | | | |
| The tutor helped me to make individual progress | | | | |
| The tutor seemed to know the topics | | | | |
| I think the tutor had prepared the problems well for discussing | | | | |
| Her/his way of interrupting was not hurting the group's discussion | | | | |
| The tutor's contributions were relevant | | | | |
| Overall, the tutor was performing her/his role well | | | | |

**Table S6. HowUlearn questionnaire**

| *Studying and learning I* | Consider your studies as a whole in your faculty or discipline, and response to the following questions and statements. Consider the most typical course (lecturing, group work etc.) which you have participated. | | |
|---|---|---|---|
| | **I completely disagree** | **I neither agree nor disagree** | **I fully agree** |
| I often have trouble making sense of the things I have to learn | | | |
| I put a lot of effort into my studying | | | |
| Much of what I've learned seems no more than unrelated bits and pieces | | | |
| On the whole, I've been systematic and organized in my studying | | | |
| Ideas and perspectives I've come across while I'm studying make me contemplate them from all sides | | | |
| I look at evidence carefully to reach my own conclusion about what I'm studying | | | |
| I am unable to understand the topics I need to learn because they are so complicated | | | |
| I organize my study time carefully to make the best use of it | | | |
| Often I have to repeat things in order to learn them | | | |
| I carefully prioritize my time to make sure I can fit everything in | | | |
| I try to relate new material to my previous knowledge | | | |
| I try to relate what I have learned in one course to what I learn in other courses | | | |

| | | | |
|---|---|---|---|
| *Studying and learning II* | | | |
| | **I completely disagree** | **I neither agree nor disagree** | **I fully agree** |
| I believe I will do well in my studies | | | |
| I'm certain I can understand the most difficult material in my studies | | | |
| I'm confident I can understand the basic concepts of my own study field | | | |
| I expect to do well in my studies | | | |
| I'm certain I can learn well the skills required in my study field | | | |
| *Development of teaching* | Consider your studies as a whole in your faculty or discipline, and response to the following questions and statements. | | |
| | **I completely disagree** | **I neither agree nor disagree** | **I fully agree** |
| It is clear to me what I am expected to learn in the courses | | | |
| What we are taught seems to match what we are supposed to learn | | | |
| I can see the relevance of what we are taught | | | |
| Students support each other and try to give help when it is needed | | | |
| I find most of what I learned in courses really interesting | | | |
| Talking with other students helps me to develop my understanding | | | |
| I enjoy participating in courses | | | |
| I can generally work comfortably with other students | | | |
| I receive enough feedback about my learning | | | |
| It is clear to me what is expected in the assessed | | | |

| | | | |
|---|---|---|---|
| work (i.e., final exam, exercises) | | | |
| I can see how the set work fit in with what we are supposed to learn | | | |
| The feedback given on my work helps me to improve my ways of learning and studying | | | |
| The set work helps me to make connections to my existing knowledge | | | |
| The feedback given on my set work helps to clarify things I hadn't fully understood | | | |
| | Consider the most typical teaching arrangements and methods in your faculty or discipline. What has been the most typical number of students in the study groups of your faculty or subject? | | |
| The most common size of a study group in your faculty/discipline is | - A small group max. 20 students<br>- A middle-sized group; approx. 20 to 50 students<br>- A large group, more than 50 students | | |
| Which assessment method has been the most typical one in your faculty/discipline? | - A written examination on set books<br>- A written examination at the end of a lecture course<br>- An essay or a written assignment<br>- A learning diary or a portfolio<br>- Written exercises<br>- Group assignments<br>- Oral presentations<br>- Continuous activities during the course | | |
| *Studying and learning III* | Please choose the alternative that best describes your situation (estimation from previous month) | | |
| - I feel overwhelmed by the work related to my studies<br>- I feel a lack of study motivation and often think of giving up<br>- I often have feelings of inadequacy in my studies<br>- I often sleep badly because of matters related to my studies<br>- I feel that I am losing interest in my studies<br>- I'm continually wondering whether my studies have any meaning<br>- I brood over matters related to my studies during my free time<br>- I used to have higher expectations of my studies than I do now<br>- The pressure of my studies causes me problems in my close relationships with others | | | |
| *General working life competences* | Consider the development of your skills during your university studies, and answer the following statements. | | |
| | **I completely disagree** | **I neither agree nor disagree** | **I fully agree** |
| I have learned to apply theoretical knowledge to practice | | | |

| | | | |
|---|---|---|---|
| My studies have developed my collaboration skills | | | |
| I have learned to analyze and categorize information | | | |
| I have learned to see things from different points of view | | | |
| I have learned to make arguments for my thoughts | | | |
| Studying at the university has developed my skills in acting as a group member | | | |
| I've learned how to make my points across in different interaction situations | | | |
| I've learned to solve problems in practical situations | | | |
